# CO_2_ Chemistry

**DOI:** 10.3762/bjoc.11.76

**Published:** 2015-05-07

**Authors:** Thomas E Müller, Walter Leitner

**Affiliations:** 1 CAT Catalytic Center, RWTH Aachen University, Worringerweg 2, 52074 Aachen, Germany; 2 Lehrstuhl für Technische Chemie und Petrolchemie, ITMC, RWTH Aachen University, Worringerweg 1, 52074 Aachen, Germany

**Keywords:** CO_2_ utilization, energy balance, reactivity, renewable resources, sustainability, value generation

It is our pleasure to introduce this Thematic Series on CO_2_ chemistry for the Beilstein Journal of Organic Chemistry (BJOC). Today’s growing demand for energy, materials and chemicals has prompted renewed interest in CO_2_ chemistry. More resource-efficient chemical processes are being implemented, while we are facing the change from a fossil fuel-based society to one that must rely on the sustainable use of renewable resources. Although there are many ways to harness renewable energy resources, much of the needed materials and chemicals will continue to be carbon-based.

**Figure 1:**
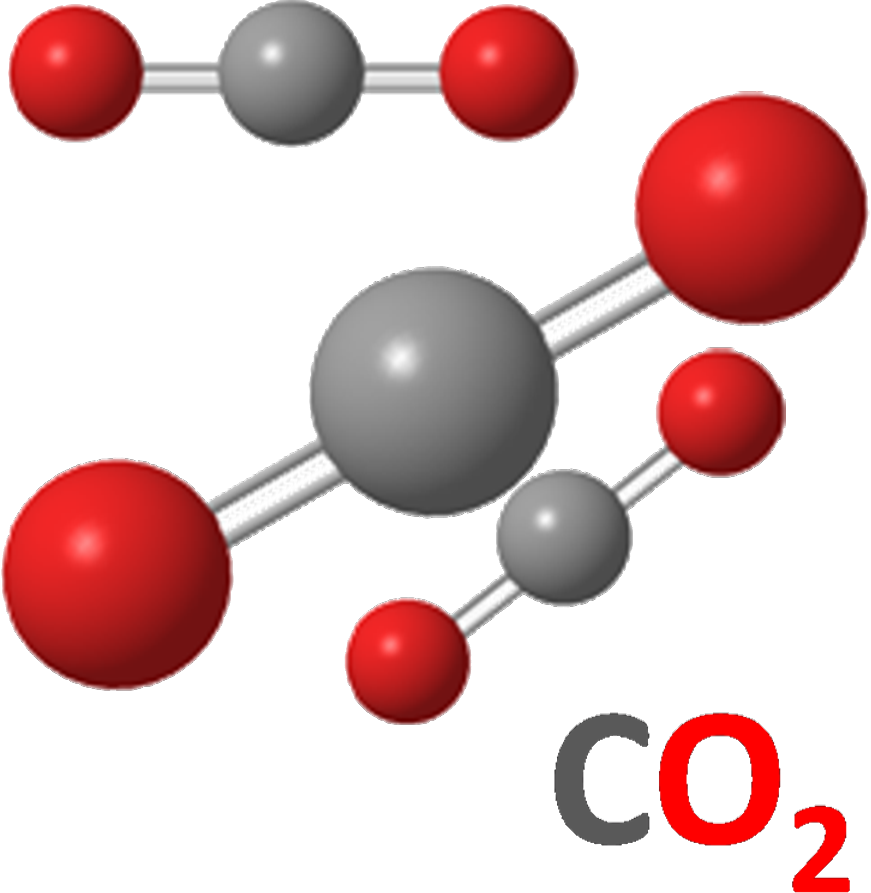
The carbon dioxide molecule.

One of the most abundant renewable resources of carbon is carbon dioxide (Figure 1). Carbon capture technologies are being implemented [1] to capture a part of the yearly anthropogenic CO_2_ emission of 36,600 million metric tons of CO_2_ [2]. If only a fraction of the captured CO_2_ stream could be made available for chemical production, a significant contribution to the annual production of carbon-based materials and chemicals could be supplied. Here, we offer the reader to relate these figures with the annual production of polymeric materials of 280 million metric tons [3]. Remarkably, 110 million metric tons of CO_2_ per year for producing urea, methanol and salicylic acid are industrial reality today. These applications clearly illustrate the path forward. Due to the abundant availability of pure CO_2_ gas streams [1], it is only logical to promote a more widespread use of carbon dioxide as chemical feedstock. Notably, the use of CO_2_ for manufacturing materials and chemicals is still in its infancy.

**Figure 2:**
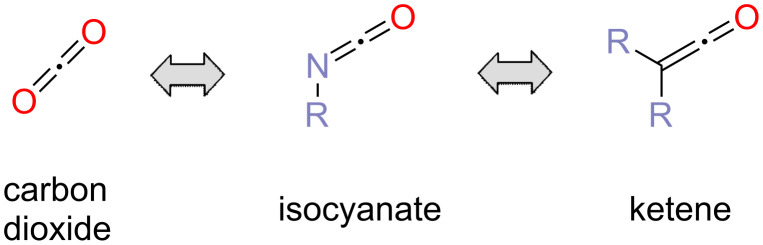
Examples of highly reactive molecules that are isoelectronic to carbon dioxide.

Carbon dioxide (CO_2_) has long stirred the fascination of chemists. A rich chemistry has evolved utilizing this molecule in chemical synthesis [4]. Hitherto the low reactivity of the CO_2_ molecule poses significant challenges to the utilization of carbon dioxide in industrial applications. Thus, the CO_2_ molecule is commonly perceived to be highly inert. This perception clearly stems from the high chemical stability of carbon dioxide. However, the reactivity of the CO_2_ molecule may be underestimated. Carbon dioxide is isoelectronic to highly reactive molecules such as isocyanates and ketenes (Figure 2). This implies that reactivity and kinetic limitations may be encountered much less frequently in the chemical conversion of carbon dioxide than generally assumed.

**Figure 3:**
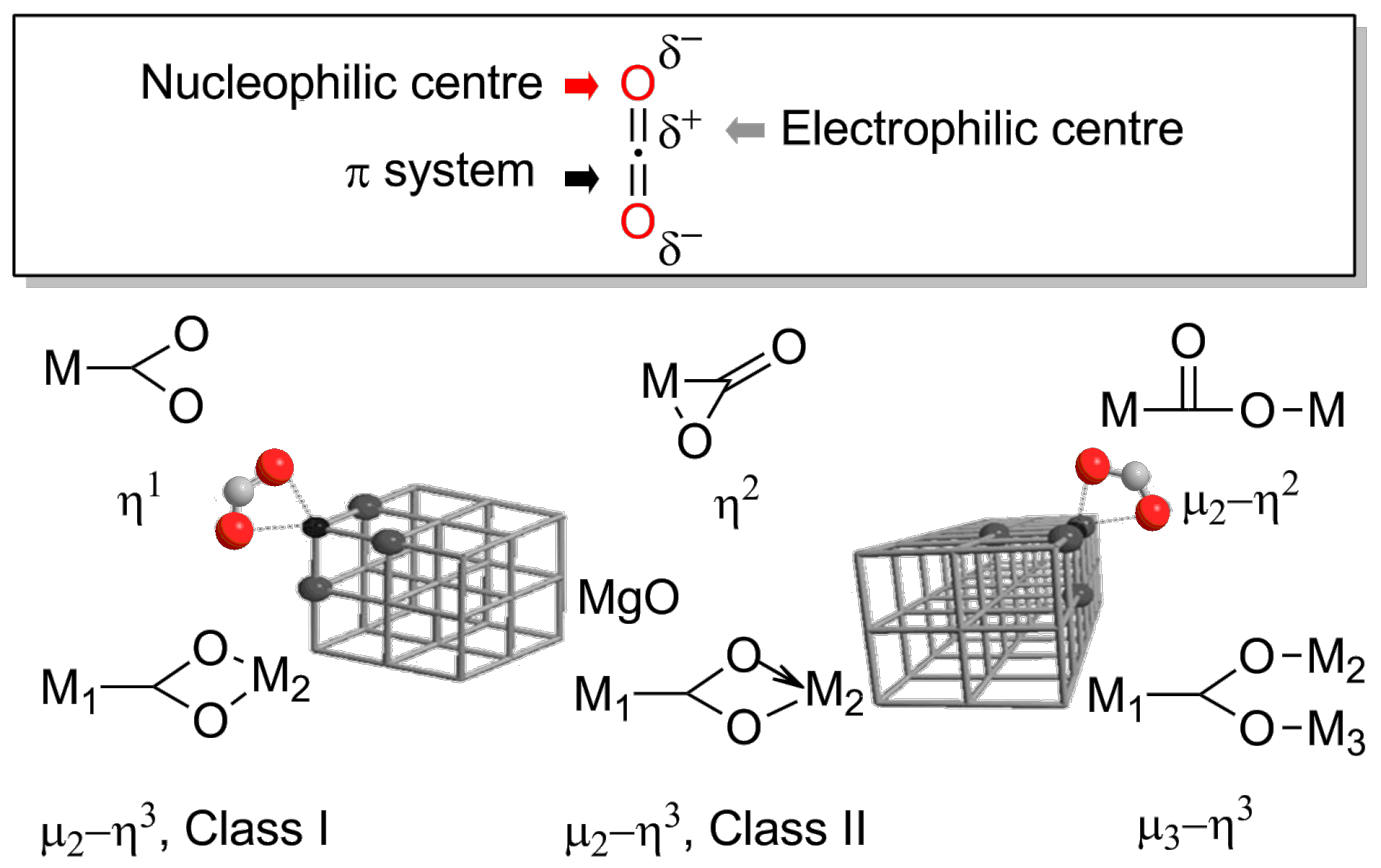
Threefold reactivity of carbon dioxide and examples for different activation modes for CO_2_ involving metal centres in homogeneous and heterogeneous catalysts [5–6].

To overcome its thermodynamically low level, additional energy is required to activate the CO_2_ molecule. The threefold reactivity (Figure 3) of CO_2_ with a nucleophilic oxygen atom, an electrophilic carbon atom and a π system provides the chemist with many options. Likewise, a rich coordination chemistry to metal centres has been reported for CO_2_ [5–6]. A forthcoming path is the reaction of CO_2_ to form energy-rich intermediates that can subsequently transfer the CO_2_ molecule to target substrates [7]. The use of efficient catalysts is often another requisite to direct the reaction pathways with high selectivity to yield the desired target products and to overcome kinetic limitations associated with certain slow elementary steps.

This Thematic Series on CO_2_ chemistry presents intriguing approaches regarding different methodologies to activate carbon dioxide. One emerging field is the electrochemical fixation of CO_2_, which can be applied in the synthesis of carboxylic acids [8]. Also highly interesting is the combination of enzymatic and photocatalytic approaches for activating CO_2_ [9]. Bifunctional catalyst systems are frequently needed and well-understood in the synthesis of cyclic carbonates [10]. Activation of carbon dioxide by inserting it into metal-alkoxide bonds allows for subsequent applications in polymer synthesis such as the copolymerisation of carbon dioxide with epoxides and other co-monomers [11]. Here, the catalysis with cobalt complexes still presents surprising effects [12]. More efficient systems for CO_2_ capture are being developed on the basis of amine-functionalised ionic liquids where zwitterionic adduct formation is the key to higher efficiency [13]. Furthermore, many physical properties of carbon dioxide are outstanding, making supercritical carbon dioxide a solvent like no other [14].

Altogether, the articles in this Thematic Series present a remarkable overview of opportunities in the field of CO_2_ chemistry from many of its top practitioners. These opportunities are harbingers of the many additional reactions, reactivity modes and catalysts that remain to be discovered. Exploiting carbon dioxide to create economic value will be the driving force for the more widespread use of this fascinating molecule. In the long term, we envision mankind creating an anthropogenic carbon loop where CO_2_ released at the end of the life span of carbon-based goods of everyday life is again employed in the production of new materials and chemicals.

We are highly grateful to the authors for their excellent contributions towards making this Thematic Series as successful as the previous editions.

Thomas E. Müller and Walter Leitner

Aachen, April 2015
